# Malignant Transformation of a Mature Cystic Ovarian Teratoma into Thyroid Carcinoma, Mucinous Adenocarcinoma, and Strumal Carcinoid: A Case Report and Literature Review

**DOI:** 10.1155/2012/269489

**Published:** 2012-09-19

**Authors:** Hilary D. Hinshaw, Ashlee L. Smith, Mohamed Mokhtar Desouki, Alexander B. Olawaiye

**Affiliations:** ^1^Department of Gynecologic Oncology, Magee-Womens Hospital of UPMC, 300 Halket Street, Pittsburgh, PA 15213, USA; ^2^Department of Pathology, Breast, and Gynecologic Pathology, Magee-Womens Hospital of UPMC, PA 15213, USA

## Abstract

Malignant transformation of a mature cystic teratoma (MCT) is an infrequent, often asymptomatic event. We report the first example of a struma ovarii with a focus of follicular variant of papillary thyroid carcinoma (a), mucinous adenocarcinoma (b), and strumal carcinoid tumor (c)—all three arising in one mature cystic teratoma of the ovary. From our reviews, we found limited data to guide management when these malignant foci occur within an MCT. Consideration should be given to thyroidectomy followed by total-body scanning and serum studies for foci of thyroid carcinoma and adjuvant therapy with thyroidectomy and radioablation if residual disease is identified (a). Additionally, extrapolating from data for mucinous adenocarcinomas, consideration could be given to adjuvant chemotherapy after appropriate staging (b). Strumal carcinoid tumors should be treated as tumors of low malignant potential. Observation is appropriate if after complete staging, no invasive implants are noted (c).

## 1. Introduction

Mature cystic teratoma (MCT) is not a rare occurrence, accounting for about 20% of ovarian tumors, but malignant transformation of MCT is infrequent [1]. Reports vary, but the risk of transformation is estimated to be between 0.17–2% and this most commonly occurs in postmenopausal women. As expected, any component of the MCT can undergo malignant transformation, but about 80% of the time squamous cell carcinoma is found [2]. Some less common malignancies include thyroid carcinomas, adenocarcinomas, and carcinoid tumors [2, 3]. Even rarer is a single MCT containing multiple types of malignant transformation.

Struma ovarii is a rare monodermal ovarian teratoma, diagnosed when a teratoma specimen contains at least 50% thyroid tissue. These represent about 2% of MCT and most commonly occur in the fifth or sixth decades of life. Malignancy within struma ovarii occurs in roughly 5%, or roughly 0.1% of MCT. Typically these malignancies are found postoperatively. These carcinomas can be classified into three types: papillary, follicular variant of papillary, and follicular [1, 4]. 

Mucinous tumors are classified as a surface epithelial tumor. However, 3–5% of such tumors develop in association with MCT. Historically, mucinous tumors have been difficult to interpret and are classified as either benign, borderline, or carcinoma. Borderline tumors can be of two types, endocervical or intestinal. Sometimes, mucinous tumors are actually metastatic to the ovary from another intra-abdominal organ. Typically, metastatic disease to the ovary is indicated bilaterally, by smaller dimension (<10 cm), and advanced stage [5]. Adenocarcinoma, which includes mucinous type, accounts for 6.8% of all malignant transformations of MCT [6].

Primary ovarian carcinoid tumors are also very rare and account for fewer than 0.1% of all ovarian carcinomas. They can be subdivided into four types: insular, strumal, trabecular, and mucinous. They may occur with or without an associated MCT, and although most commonly as a singular subtype, mixed primary ovarian carcinoids have been reported [7]. Presenting symptoms, if present, can include carcinoid syndrome and pressure or pain with defecation. The insular type is most common and this is the only type that has been associated with the carcinoid syndrome [8]. Strumal carcinoid, the second most commonly observed subtype, is characterized by a mixture of carcinoid and thyroid tissue, and thought to be of endodermal origin with thyroid and C-cell differentiation [7]. Strumal carcinoids have been associated with virilism as well as postoperative thyroid storms and hypothyroidism [9, 10].

Forthcoming is a case report of a postmenopausal patient with a cystic and solid right ovarian mass. The mass was found to be a mature cystic teratoma containing three separate and different malignant foci.

## 2. Case Report

A 74-year-old, gravida1 para1 Caucasian female presented following an incidental finding of a pelvic mass noted on CT scan completed for the evaluation of postoperative edema. The patient recently underwent open heart surgery with two valve replacements approximately two months prior to presentation at our center. Postoperatively she experienced generalized edema and a workup to elucidate the underlying cause was significant for a CT scan demonstrating a 13 × 9 cm right adnexal mass with fat, soft tissue, and bony elements, consistent with a teratoma. Notably, the CT was negative for adenopathy or omental caking. Subsequent ultrasounds corroborated these findings and additionally noted an endometrial lining of 7 mm. Except for the above mentioned edema, her review of systems was entirely negative. Her medical history is significant for coronary artery disease, atrial fibrillation, congestive heart failure, hypertension, and type 2 diabetes mellitus. 

The patient underwent a robotic assisted total laparoscopic hysterectomy with bilateral salpingo-oophorectomy (BSO), bilateral pelvic and para-aortic lymph node dissection, infracolic omentectomy, and staging biopsies. Intraoperative pathology suggested a portion of the right ovary contained malignant cells, suggestive of thyroid carcinoma. There was no gross evidence of metastatic disease. 

**Figure 1:**
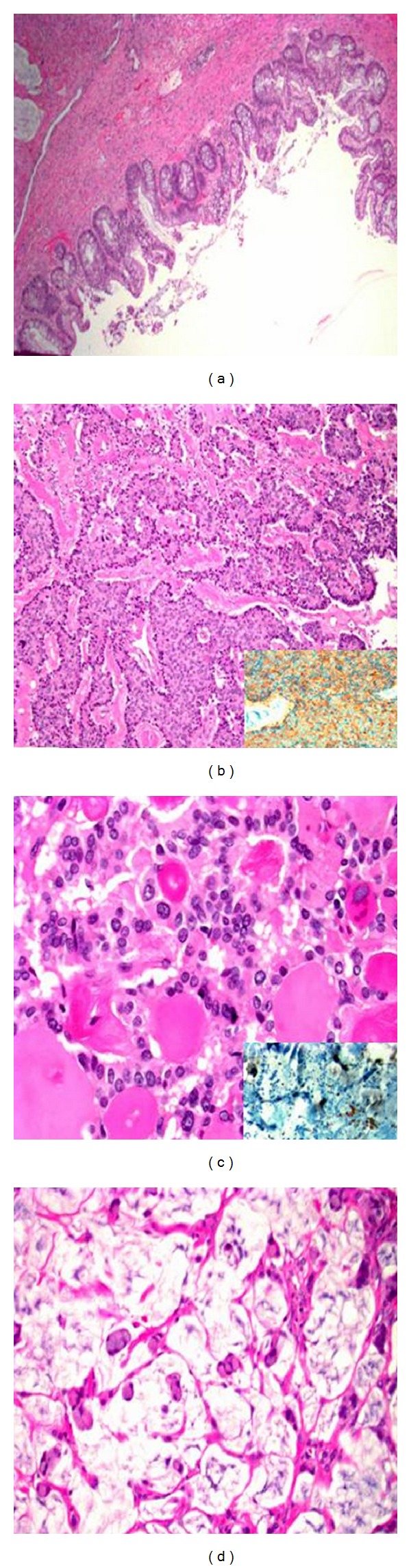
Malignant components in the teratoma. (a) Benign large bowel component of mature cystic teratoma. (b) Strumal carcinoid, strongly positive for synaptophysin stain (inset); (c) follicular variant of papillary thyroid carcinoma with positive nuclear staining for TTF1 (inset). (d) Infiltrating mucinous adenocarcinoma.

Pathologic gross examination revealed a previously ruptured, right ovarian mass weighing 143 grams that measured 8.5 × 7.3 × 5.5 cm. The outer surface was pink-white, hyperemic and smoothly bosselated. On opening, the mass was cystic, filled with grumous tan-brown material and had a raised, 2.5 × 2.0 × 1.5 cm protrusion and multiple solid tan-yellow to tan-white tissue with minimal hemorrhage. A minimal amount of hair was also identified. No normal appearing ovarian parenchyma was seen grossly. Two representative portions are submitted for frozen section evaluation. Additionally, eight more sections submitted for permanent diagnosis. Intraoperative consultation revealed cells with high nuclear cytoplasmic ratio, chromatin clearing, pseudoinclusions suggestive of malignant tumor arising in teratoma possibly thyroid carcinoma. Microscopic evaluation of permanent sections demonstrated a mature cystic teratoma with struma ovarii component arising from the right ovary with multiple malignant foci. The mature cystic teratoma components consist of skin, cartilage, benign colonic, and thyroid tissues (Figure 1(a)). The malignant components consist of (1) well differentiated strumal carcinoid tumor confirmed with positive immunohistochemistry (IHC) for synaptophysin (Figure 1(b)), (2) well differentiated follicular variant of papillary thyroid carcinoma arising in struma ovarii tested positive for Thyroid Transcription Factor 1 (TTF1) (Figure 1(c)), Thyroglobulin and Cytokeratin 7 (CK7) by IHC, and (3) moderately differentiated mucinous adenocarcinoma arising in intestinal type mucinous cystadenoma (Figure 1(d)) which tested negative for CK7, negative for CDX2 and positive for CK20 by IHC. Additional findings included a benign mature cystic teratoma of the contralateral left ovary and adenomyosis. All lymph nodes, peritoneal biopsies, the infracolic omentum, and washings were negative for metastatic disease. Thus, the TNM staging rendered FIGO stage IC.

## 3. Literature Review

### 3.1. Malignant Struma Ovarii (a)

A Medline search using keywords malignant struma ovarii and teratoma revealed numerous case reports as well as two recent reviews [1, 4]. The reviews noted average ages of 42.9 and 44 years and pain as the most common presenting symptom. Pooled recurrence rates were reported as 15 and 38%. Recurrences were discovered as late as 16 years after hysterectomy and BSO, but average time to recurrence was 4 years. A 2002 review included 24 patients: 16 patients without and 8 patients with adjuvant treatment, most commonly thyroidectomy and ^131^I. One of the 24 patients experienced persistent disease postoperatively, but 8 of 23 recurred after complete response to initial surgery. All eight recurrences were noted in patients who did not receive adjuvant therapy. No recurrences were noted in the group of seven patients receiving adjuvant therapy after complete response to initial surgery [4].

### 3.2. Mucinous Adenocarcinoma Arising in Intestinal Type Mucinous Cystadenoma (b)

A Medline search using combinations of keywords mature teratoma and intestinal type and mucinous and glandular epithelial neoplasms returned little data applicable to our patient. In one case series of 42 patients with mucinous epithelial tumors arising in association with mature ovarian teratomas, 12% (*n* = 5) were classified as mucinous carcinoma and 40% were classified as mucinous cystadenoma. Three of five carcinomas had peritoneal carcinomatosis and classical pseudomyxoma peritonei noted during initial surgery. Treatment was not discussed, and follow up was only available for the aforementioned three. One died of disease at 6 months, and two were alive with disease, one immediately after surgery and the second at 29 months [11]. 

Three case reports were also found. The first, a 62-year-old female who underwent a hysterectomy and BSO for a 35 cm left ovarian mass, was significant for a mucinous adenocarcinoma, likely of gastrointestinal origin, within a MCT. There was no evidence of metastatic disease at the time of surgery. She was treated in 1976 with chemotherapy and was without evidence of disease 15 years later [6]. The second, a 45-year-old female who underwent a total vaginal hysterectomy and BSO for benign indications had an incidental finding of ovarian cysts with focal intestinal-type epithelium with focal malignant transformation to intestinal-type adenocarcinoma. This carcinoma did not invade surrounding benign ovarian parenchyma. Without adjuvant therapy, she was alive and well 5 years following her surgery [12]. The third, a 62-year-old female who underwent a hysterectomy, BSO and Hartmann's operation was significant for pathology of ovarian mucinous cystadenocarcinoma and strumal carcinoid arising in an MCT and invasive cervical carcinoma. At the time of surgery there was no evidence suggestive of malignancy. Postoperatively she received three cycles of cisplatin and paclitaxel (Taxol), as well as pelvic radiation. Her CA 19-9 and CEA normalized and she was without evidence of disease at 13 months. The authors suggested CA 19-9 may be useful in monitoring such mucinous tumors. They also suggested carboplatin and Taxol, extrapolating from data on ovarian epithelial cell carcinoma. They were in agreement with others' recommendations to treat strumal carcinoid with salpingo-oophorectomy [3].

### 3.3. Strumal Carcinoid Tumor (c)

A Medline search using keywords strumal carcinoid revealed several case series, but none from the past decade. A case series of 50 strumal carcinoids published in 1980 included only one patient who died of her disease. A 53-year-old white female presented with abdominal swelling. Intra-operatively, a 16 cm right ovarian tumor was noted, and on final pathology, 75% of her mature teratoma was strumal carcinoid. Post-operatively, she developed thyroid storm. After developing abdominal pain and weight loss, a recurrence was diagnosed 1.5 years later. Thiotepa (ThioTEPA) was given, but the patient died of her disease one year after diagnosis of recurrence. Interestingly, although this patient was diagnosed with a strumal carcinoid, only the carcinoid element metastasized. In this same case series, five patients were deceased from other causes, but 90% of patients were alive and well [10]. These authors concluded the tumor is almost always benign and can be treated with an oophorectomy. 

A second review of 150 patients with primary ovarian carcinoids published in 1984 also concluded that these metastasize only occasionally and should be treated as ovarian tumors of low malignant potential [8].

Subsequent to these reviews, Armes reported on a 24-year-old pregnant female presenting with torsion who was found to have widespread metastases of a strumal carcinoid. This patient's pathology, interestingly, was reported as more reminiscent of a malignant struma ovarii, as only the thyroid follicular carcinoma component was noted to be metastatic. The pregnancy was terminated, and she was initially treated with 3 cycles of bleomycin, etoposide (VP-16), and cisplatin. This was unsuccessful, but further treatment with thyroidectomy and ^131^I resulted in regression of metastatic disease [13]. 

A third review of 17 primary ovarian carcinoid tumors published in 1996 included four not previously reported primary ovarian carcinoids with strumal histology treated with cystectomy (one) or hysterectomy and BSO (three). All were stage I at presentation and none had evidence of disease at 2 or more years from diagnosis. In addition to the new patients, a literature review was also included which was notable for 32 patients with strumal carcinoid with MCT. All were stage I at presentation with only one patient (or 3%) died of her disease. For the 17 new patients, five-year survival was excellent (100%) when disease was confined to one ovary (11 of 17). One patient recurred at 13 years and died of disease at 14 years. The 6 patients with stage III or IV disease all had insular histology, and their five-year survival was reported as 33%. The authors concluded that primary ovarian carcinoid tumors treated with surgery alone and found to be confined to the ovary are expected to have an excellent outcome [7].

## 4. Discussion

### 4.1. Malignant Struma Ovarii (a)

Likely due to the rarity and resulting inability to design management trials, there is no consensus on the treatment of malignant struma ovarii. Various case studies recommend different treatment options. Surgical treatment ranges from unilateral oophorectomy to complete staging. Adjuvant treatment possibilities include radiation, traditional chemotherapy, thyroidectomy and ^131^I ablation, and thyroxine suppression therapy [4, 14]. Some advocate a post-operative total-body ^131^I scintiscan to assess for residual malignant disease and trending serum thyroglobulin levels to monitor for recurrence and adjuvant therapy only if these were positive, and this should be used with caution if not in the setting of thyroidectomy as most malignant struma ovarii have poor iodine uptake and thyroid hormone synthesis [1, 15]. The rationale for each treatment is based in a balance of risks and complications of adjuvant therapy versus potential for recurrence—recurrence that carries a significant risk of death from disease. One option to guide postoperative treatment is risk stratification, a concept that has its basis in the treatment of thyroid carcinoma. A low risk carcinoma is defined as being confined to the struma ovarii, less than 2 cm, and without poorly differentiated histologic features. Low risk patients could be given thyroxine, with a target serum TSH between 0.1 and 0.5 mIU/L, which is a similar TSH suppression goal used in the treatment of thyroid carcinoma. These patients should then have periodic thyroglobulin measurements and any rise should prompt thyroidectomy, radioiodine imaging and treatment as indicated [16]. Patients not receiving adjuvant thyroidectomy and ablation should be counseled on the risk of recurrence, estimated between 15 and 38%, and the need for close followup and monitoring as recurrences have been noted to occur more than a decade from the initial diagnosis.

As expected, treatment is even more complex when future fertility is desired. Although not applicable to our patient, if fertility is desired consideration could be given to retaining the uterus and contralateral ovary when there is no evidence of extra-ovarian spread during staging surgery. Post-operative management could include suppressive treatment with thyroxine.

### 4.2. Mucinous Adenocarcinoma Arising in Intestinal Type Mucinous Cystadenoma (b)

There is little information on treatment outcomes with mucinous adenocarcinoma within MCT without evidence of metastatic spread. One proposed approach is to treat this disease as one would treat primary ovarian mucinous type epithelial carcinomas. A second approach is expectant management without further surgical staging [12]. A post-operative discussion including the rarity and inadequate data on treatment is imperative. A risk and benefit discussion should be had with the patient, keeping in mind her age, comorbidities, surgical, and pathological findings.

### 4.3. Strumal Carcinoid Tumor (c)

All of the reviewed literature suggests that stage I strumal carcinoid tumors can be treated with surgical excision. Most commonly this is done by salpingo-oophorectomy (unilateral or bilateral, with or without hysterectomy), but there are reports of 5 year disease free survival with cystectomy alone [7, 10]. Metastatic disease seems to be limited to either the carcinoid or thyroid element, no reports of dual metastases were found. The single report of the metastatic thyroid element was successfully treated with thyroidectomy and ^131^I ablation, similar to the treatment of metastatic struma ovarii [13]. The single report of the metastatic carcinoid element was unsuccessfully treated with ThioTEPA [10]. 

In summary, this is a very unique patient with multiple malignant transformations within one MCT. Six months postoperatively, she is doing well and has no evidence of disease. After a multidisciplinary meeting and then discussions with the patient and her family, keeping in mind her medical comorbidities, surgical findings, pathology, and wishes, no further surgery or chemotherapy is planned. For the component of malignant struma ovarii, a ^131^I total-body scintiscan was done, recognizing the limitations of this study, and did not show any extrathyroidal functioning tissue. For her mucinous adenocarcinoma and stromal carcinoid elements, it was decided that surgical resection was sufficient treatment. We will continue to follow her every three months for clinical evidence of disease.
